# Where do PDD and DLB SYNdromes fit in neuronal alpha-SYNuclein biological frameworks?

**DOI:** 10.1007/s00702-025-03060-5

**Published:** 2025-12-04

**Authors:** David J. Irwin

**Affiliations:** 1 LBDA Research Center of Excellence, Department of Neurology, University of Pennsylvania Perelman School of Medicine, Philadelphia, USA; 2 Penn Frontotemporal Degeneration Center, Department of Neurology, Perelman School of Medicine, University of Pennsylvania, Philadelphia, USA; 3 Penn Digital Neuropathology Lab, Department of Neurology, Perelman School of Medicine, University of Pennsylvania, Philadelphia, USA; 4 Department of Neurology, University of Pennsylvania Perelman School of Medicine Hospital of the University of Pennsylvania, 3600 Spruce Street, Philadelphia, PA 19104 USA

**Keywords:** Parkinson’s disease, Dementia with lewy bodies, Neuronal alpha-synuclein, Alzheimer’s disease, Biological classification

## Abstract

Lewy body disorders (LBD) are a spectrum of neurodegenerative diseases characterized by the presence of misfolded neuronal alpha-synuclein (aSYN) pathology in the central and peripheral nervous system. LBDs have heterogeneous clinical presentations, which include dementia with Lewy bodies (DLB), Parkinson’s disease (PD), and PD with dementia (PDD). Thus, LBD clinical syndromes (PD/PD/DLB) represent clinicopathologic entities (i.e. constellations of symptoms and supportive biomarkers with a high specificity for underlying aSYN pathology), but clinical features between PDD and DLB largely overlap. Indeed, there is longstanding debate over the utility of the clinical designation between PDD and DLB due to shared underlying pathology, genetic risk factors and prodromal features. Recent advances in the ability to detect pathological aSYN from peripheral fluids/tissues in living patients has ushered in a new era of biological classification of LBD, providing opportunity to improve antemortem diagnosis and facilitate disease-modifying therapeutic trials. The clinical interpretation of these and future aSYN-specific biological tests will be complex, and the reconciliation of classic LBD syndromes with emerging biological classification schemes for LBD and other neurodegenerative disorders is a priority. Indeed, varying burden of aSYN is also found postmortem in > 50% of clinical Alzheimer’s disease (AD), and to a lesser frequency as co-pathology in other neurodegenerative disorders, and incidentally in adults without neurologic disease. This review summarizes autopsy-confirmed data on the clinical expression of LBDs and the boundaries between PDD, DLB and mixed-pathology AD to inform the interpretation of emerging biological tests for aSYN and biological classification schemes for LBD and AD.

## Introduction

### Clinicopathologic syndromes of PDD and DLB

Neurodegenerative disorders historically used clinicopathologic terms for diagnosis, where a set of core clinical criteria to define the patient’s neurologic signs and symptoms are synonymous with a single underlying neuropathology. Indeed, Alzheimer’s dementia (AD) has been clinically defined as an age-associated slowly progressive multidomain amnestic syndrome corresponding to amyloid-beta (AB) plaques and tau-positive neurofibrillary tangles (NFTs) postmortem (McKhann et al. 2011) (i.e. Alzheimer’s disease neuropathologic change; ADNC (Hyman et al. 2012). With the advent of advanced histopathologic techniques to visualize the high frequency of additional brain pathologies in aging (Robinson et al. 2023; Schneider et al. 2007, 2009), and clinically-available diagnostics to detect amyloidosis (Klunk et al. 2004; Clark et al. 2012; Shaw et al. 2011) and NFTs (Fleisher et al. 2020) in vivo, there is increasing recognition of the complexity between clinical features of neurodegenerative dementias and underlying brain pathology, necessitating a biological approach (Jack et al. 2016, 2024). This is of particular importance for Lewy body disorders (LBDs), including Parkinson’s disease (PD), PD with dementia (PDD) and dementia with Lewy bodies (DLB), which have the shared underlying neuropathological substrate of misfolded neuronal alpha-synuclein (aSyn; i.e. synucleinopathies), but partially-overlapping clinical heterogeneity that is poorly understood (Lippa et al. 2007; Irwin et al. 2013). Moreover, there is clinical and pathological overlap of LBDs with AD (Coughlin et al. 2020a), adding further complexity to accurate diagnosis and biological classification of these disorders. The advent of seeding amplification assays (SAAs) for a-Syn from cerebrospinal fluid (CSF) (Fairfoul et al. 2016; Groveman et al. 2018) and other tissues (Manne et al. 2020; Mammana et al. 2021), as well as histopathologic detection of pathologic a-Syn in peripheral nerves from skin biopsies (Gibbons et al. 2022, 2024; Chahine et al. 2020) (reviewed in detail elsewhere (Coughlin and Irwin 2023; Scott et al. 2021), has for the first time facilitated an in vivo biological diagnosis of underlying aSyn. While these tests currently are not fully validated for clinical use, in the research setting they are an important advance to potentially improve biologic characterization for clinical research and trials for LBDs. Thus, recent efforts to develop biological definitions for LBD have been proposed (Höglinger et al. 2024; Simuni et al. 2024a) and emerging biological definitions of AD now incorporate biomarker evidence of aSyn co-pathology (Jack et al. 2024). The complexity of clinicopathological correlations in neurodegenerative disease and emerging biological tests of underlying brain pathology motivate an evolving nomenclature (Dementia Nomenclature Initiative) that propose a new framework to categorize clinical features and separately link these to available biologic data to characterize the underlying etiology rather than a clinicopathologic diagnosis alone (Petersen et al. 2023). Considering these paradigm shifts, here the shared and disparate features of DLB and PDD clinicopathologic syndromes from autopsy-based studies are reviewed to inform future potential approaches of clinical and biological characterization across LBDs and AD.

The clinical syndrome of DLB is critically important to diagnose and differentiate patients with likely underlying aSyn from patients with AD and other neurodegenerative diseases (McKeith et al. ,^1996^ , ^1996^, ^2005^, ^2017^ ). Indeed, early postmortem observations of neocortical Lewy bodies (LBs) using hematoxylin and eosin stained tissue, together with clinical observations of patients with disproportionate non-amnestic cognitive impairments often with neuropsychiatric features of spontaneous persistent well-formed visual hallucinations and fluctuations in mentation and/or motor Parkinsonism, suggested a distinct non-AD dementia with a neuropathologic substrate of cortical Lewy pathology with or without mixed ADNC pathology (McKeith et al. 1992;1996 Hansen et al. 1990; Woodard 1962; Perry et al. 1990; Kosaka et al. 1976). The discovery of mutations in the *SNCA* gene encoding a-Syn in families with Parkinson’s disease (PD) with LB pathology (Polymeropoulos et al. 1997), along with postmortem findings of aSyn as the core protein aggregation within LBs and Lewy neurites (LNs) (Spillantini et al. 1997), helped to establish the central role of aSyn in the pathogenesis of both PD and DLB. Moreover, this also led to modern immunohistochemical techniques to detect pathologic aSyn in LBs and LNs with high sensitivity in non-motor brain regions which enhances clinicopathologic studies to further refine DLB criteria. Indeed, the most recently revised clinical criteria for DLB (McKeith et al. 2017) includes supportive biomarkers that each have autopsy validation for indirect in vivo measurement of aSyn degeneration in the striatonigral system (dopamine transporter imaging) (Colloby et al. 2012; Thomas et al. 2017), sympathetic cardiac denervation (myocardial scintigraphy) (Orimo et al. 2005) and polysomnogram-confirmed rapid-eye movement sleep behavior disorder (REM SBD) (Dugger et al. 2012).

In parallel, longitudinal natural history studies of PD patients increased the recognition of non-motor features of the disease, particularly cognitive impairment, where it is now estimated that many PD patients will eventually develop dementia during the disease course (Aarsland et al. 2003; Hely et al. 2008; Cummings 1988). Moreover, mild-cognitive impairment (MCI) that does not meet the threshold of functional impairment for a dementia in PD is common, including ~ 25% of PD patients at the time of diagnosis (Muslimovic et al. 2005). Thus, “PD/PDD” here will represent the evolution of PD to PDD in most patients. The development of formal diagnostic criteria for PDD (Emre et al. 2007) and PD-MCI (Litvan et al. 2012) helped characterize the specific cognitive impairments observed in PD patients, which are heterogenous. While it is recognized that many patients with PDD have clinical neuropsychiatric features of visual hallucinations (Pagonabarraga et al. 2016) and cognitive fluctuations (Serrano and Garcia-Borreguero 2004) as seen in DLB, the onset of dementia in PD varies considerably between individual patients, with some having early manifestation of cognitive impairment compared to other PD patients with 1–2 decades or more of motor PD prior to development of dementia (Irwin et al. 2013); Hely et al. 2008; Aarsland et al. 2003; Sabbagh et al. 2009; Ballard et al. 2006). Thus, DLB clinical criteria utilize the “one-year rule” of the presence of 12 months or more of established PD prior to the onset of dementia to differentiate PDD from DLB, where cognitive impairment may precede or present without emergence of Parkinsonism in the disease course (McKeith et al. 1996). Table 1 compares the current clinical diagnostic criteria for DLB (McKeith et al. 2017) and PDD (Emre et al. 2007) which encompass various cognitive, neuropsychiatric, autonomic and motor features that are highly heterogenous and largely overlapping.

**Table 1:** Diagnostic criteria for PDD vs. DLB

	Dementia with Lewy Bodies	Parkinson’s disease Dementia
Essential features	Dementia **before or < 1 year after motor Parkinsonism** (attention, executive, visuospatial > memory, language)	Dementia **in setting of established PD (> 1 year)** (attention, executive, visuospatial, **memory retrieval** > **memory encoding**, language)
Core features	Cognitive fluctuations Visual hallucinations Spontaneous Parkinsonism *(> 1 feature)* *REM sleep behavior disorder*	
Associated/Suggestive features	*Severe neuroleptic sensitivity* Postural Instability *Repeated falls* *Syncope/Transient loss of consciousness* *Autonomic dysfunction* Depression Hallucinations in other modalities Systematized delusions	*Apathy* Depression/anxiety Hallucinations Delusions Excessive daytime sleepiness
Indicative biomarkers	*Low dopamine transporter uptake* *Reduced cardiac scintigraphy uptake* *Sleep polysomnogram-confirmed REM sleep behavior disorder*	

Data adapted from DLB Clinical Criteria (McKeith et al., Neurology 2017) and PDD Clinical Criteria (Emre et al., Mov Disorders 2007). Bold/underline = explicitly different between criteria; italics/underline = not explicitly mentioned in both criteria but known shared clinical/biomarker features

This distinction has generated debate due to the shared biological features of PDD and DLB (Lippa et al. 2007; Jellinger and Korczyn 2018; Postuma et al. 2015; Boeve et al. 2016; Jellinger 2024; Richard et al. 2002; Borghammer et al. 2024). Indeed, these syndromes are helpful to direct clinical care and may uncover new biological factors that contribute to the varying expression of cognitive and motor symptoms across LBDs. Moreover, different contexts, such as clinical care, may favor diagnostic “splitting” between PDD and DLB, whereas epidemiological and biological studies of aSyn disease, may benefit from diagnostic “lumping” of these disorders. Table 2 summarizes the main shared and disparate features of PDD vs. DLB reviewed in the following sections. Finally, AD co-pathology is one biologic factor which has a strong clinical influence and association with the distribution of aSyn in the neuroaxis on the heterogeneity of both PD/PDD and DLB (Irwin et al. 2013) ; Coughlin et al. 2020a) which is reviewed below as well. Indeed, with currently available clinical tests to detect ADNC in vivo, ADNC can be an important factor to stratify LBD patient populations (Goldman et al. 2020; Toledo et al. 2023), particularly for therapeutic trials and clinical research studies.

**Table 2:** Shared and disparate biological features of PDD vs. DLB

Biologic features supporting splitting PDD vs. DLB into syndromes.	Biologic features supporting lumping PDD vs. DLB into a spectrum of *n*-aSyn.
Varying natural history of the timing motor-dementia symptoms in PDD vs. DLB.	Lack of individual-level diagnostic accuracy for pathologic/genetic factors to distinguish PDD from DLB.
Possible divergent epicenters and topographical spread of aSyn pathology (i.e. relative sparing of the striatonigral system in a subset of DLB and incidental LBD).	Shared variable prodromal features (e.g. REM SBD, anosmia, autonomic dysfunction, neuropsychiatric, cognitive, motor).
	Shared pathogenic variants (e.g. *SNCA*) with PD/PDD and DLB at times within the same family and shared risk variants (e.g. *GBA*, *SNCA*), for both PD/PDD and DLB.

### Distinct features that support diagnostic splitting of PD/PDD and DLB as distinct clinicopathologic entities

Perhaps the most notable biological difference between PDD and DLB, is the natural history of a long time-interval from onset of motor features of PD to dementia (i.e. motor-dementia interval, MDI) in many PDD patients. The presence of cognitive impairment in PD is associated with a poor prognosis, as combined motor and cognitive impairments have an added contribution to functional impairments (Rosenthal et al. 2010; Lo et al. 2009). Moreover, the onset of dementia in PD is associated with a stereotypic decline with death on average of about 3 years from onset (Kempster et al. 2010). Thus, the clinical detection of cognitive impairment of PD is essential for the clinical care of patients and the implementation of symptomatic therapies and supportive care. Over half of patients with PD may have very long MDI (over 15 years) (Hely et al. 2008) with mean MDI of ~ 10 years on average (Aarsland and Kurz 2010). Moreover, a more recent analysis in two large PD cohorts finds dementia may occur somewhat later in the disease course, with ~ 50% of patients developing dementia 15 years after the diagnosis of PD (Gallagher et al. 2024). Thus, overall PD/PDD has a distinct natural history compared to autopsy-confirmed DLB patients, of whom have an average disease duration of ~ 6.5 years and ~ 20% of which may never develop motor Parkinsonism (Irwin et al. 2017). Therefore, for clinical care and for patient advocacy, this distinction is very useful. For example, PD-related motor symptomatic therapies such as carbidopa-levodopa may be less effective in ~ 30% of DLB patients (Molloy et al. 2005). Indeed, the atypical cognitive and neuropsychiatric features of DLB, which often require a subspecialist expertise to diagnose, often result in long diagnostic delays (Galvin et al. 2010). Therefore, the patient and caregiver experience can be vastly different between these two clinical presentations of LBD. Effective outreach materials for diagnosis and management through the DIAMOND Lewy program (O’Brien et al. 2021) in the UK, and now US, have been established, including consensus recommendations for management (Taylor et al. 2020) which is complex and often requires careful balance between pharmacological approaches to the various clinical symptoms and potential medication side effects in both PDD and DLB. Clinicopathologic data in autopsy-confirmed patients can potentially inform the biological basis for these often-discrepant natural history findings between PDD and DLB.

The neuropathological substrates of dementia in PDD include many potential age-associated pathologies that alone or in combination could contribute, including distribution of aSyn pathology beyond the striatonigral system to limbic/neocortices, ADNC co-pathology, cerebrovascular disease (CVD), subcortical cholinergic loss, limbic TDP-43, and other age-related pathologies (Jellinger et al. 2002; Jellinger and Attems 2008; Irwin et al. 2012, 2013; Halliday et al. 2011; Hurtig et al. 2000; Compta et al. 2011; Kovari et al. 2003; Mattila et al. 2000; Smith et al. 2019; Martin et al. 2023; Horvath et al. 2013; Perry et al. 1985; Whitehouse et al. 1983; Kalaitzakis et al. 2008; Jellinger 2007; Kotzbauer et al. 2012; Hughes et al. 1993; Sabbagh et al. 2009; Lashley et al. 2008; Nakashima-Yasuda et al. 2007; Uemura et al. 2022). Comparative autopsy studies between PD patients who did not develop dementia during life compared to those with PDD largely find a strong correlate of higher neocortical burden of a-Syn with the outcome of dementia in PD (Irwin et al. 2012; Martin et al. 2023; Smith et al. 2019; Horvath et al. 2013; Mattila et al. 2000; Kovari et al. 2003; Jellinger 2007; Compta et al. 2011; Hurtig et al. 2000; Halliday et al. 2011). These data generally support the staging system proposed by Braak and colleagues, of a caudal-rostral spread of aSyn from the olfactory bulb and/or medulla to ascending limbic and neocortical regions as a major correlate of dementia in PD (Braak et al. 2003; Del Tredici et al. 2002). Further, animal- and cell-model studies strongly support the cell-to-cell mechanism of spread of pathogenic aSyn species as likely central to disease pathogenesis in LBD (Luk et al. 2009, 2012a, b; Volpicelli-Daley et al. 2011; Guo and Lee 2014; Kim et al. 2019). Thus, the findings of a generally restricted distribution of aSyn to subcortical structures in PD patients without dementia and PDD with long MDI, whom often have relatively “pure” aSyn pathology (Irwin et al. 2012, 2017), suggests that perhaps there are patient and/or disease-specific factors, such as strains of pathogenic aSyn (Guo et al. ; Sun et al. 2025) and/or co-pathologies, that contribute to relative vulnerabilities of non-motor cortices and often divergent natural histories in PD/PDD vs. DLB.

While the caudal-rostral model of aSyn progression is critically important, there are likely several different complex topographical patterns of spread across the LBD spectrum. Indeed, exceptions to the caudal-rostral hypothesized spread of aSyn in PD have been described, including an olfactory-only and amygdala-predominant limbic pattern without/limited involvement of the striatonigral system (Adler et al. 2019; Beach et al. 2009; Leverenz et al. 2008; Jellinger^2008^ ) (more common in the presence of ADNC co-pathology as discussed in detail below), now unified with DLB histopathologic staging (McKeith et al. 2005, 2017) into a consensus neuropathologic diagnostic approach for Lewy-related pathology (Attems et al. 2021). Thus, these divergent patterns of aSyn could potentially help substantiate the distinction between PD/PDD and DLB. As aforementioned, a sizable minority of patients with autopsy-confirmed DLB do not have motor Parkinsonism during life (Irwin et al. 2017), including some without in vivo evidence of dopamine-transporter imaging deficits (Thomas et al. 2017; Colloby et al. 2012), suggesting an altered progression of aSyn in a subset of DLB compared to PD/PDD. Finally, rare cases of incidental Lewy body disease in the limbic/neocortex of neurologically normal individuals could also suggest an alternative progression pattern of aSyn in some patients likely to develop DLB (Frigerio et al. 2011) compared to the more common pattern of brainstem aSyn in incidental LBD autopsies, thought to be a prodromal state of PD (Dickson et al. 2008). Application of modern staging (Attems et al. 2021) and statistical modeling of aSyn progression from autopsy data in large cohorts find varying patterns based on phenotype, including more relative limbic patterns in DLB particularly with high ADNC co-pathology, compared to PD/PDD, but these clinical associations are not absolute and reflect more of a spectrum (Mastenbroek et al. 2024; Toledo et al. 2016; Kok et al. 2021; Colloby et al. 2025).

In summary, the varied natural history of motor vs. cognitive impairments in PDD vs. DLB suggest a different patient experience for many, particularly in early-stage disease. Moreover, these potential discrepant patterns of aSyn in a subset of DLB patients compared to PD may substantiate a clinicopathologic dichotomy of PD/PDD and DLB; however, the following section will review direct comparisons of PDD and DLB which also reflect significant heterogeneity both within PD/PDD and within DLB clinical syndromes.

### Shared features that support diagnostic lumping of PD/PDD and DLB as neuronal alpha-synucleinopathies

Direct comparisons of autopsy data between PDD and DLB patients vary in terms of sample size and patient characteristics, but overall find greater overall neocortical aSyn, AB and tau NFT cortical pathology, as well as greater striatal amyloidosis and aSyn in DLB vs. PDD (Ballard et al. 2006; Irwin et al. 2012; Jellinger 2004, 2007; Iseki 2004; Harding and Halliday 2001; Colosimo et al. 2003; Richard et al. 2002; Duda et al. 2002a). Increased cerebral amyloid angiopathy has also been found in DLB vs. PDD (Walker et al. 2024; Hansen et al. 2021; Jellinger 2007). While a neocortical distribution of aSyn is commonly associated with dementia in both PD/PDD and DLB, patients with a relatively “pure” limbic-predominant distribution and limited ADNC co-pathology can also present with clinical DLB or PDD, and these patients may have a slower progression compared to LBD with a neocortical distribution, often found with mixed pathology (Ferman et al. 2018, 2020). Indeed, aSyn, tau and AB pathologies are highly correlated with each other across PD/PDD and DLB (Irwin et al. 2012, 2017; Coughlin et al. 2019). This potential synergy of mixed pathology is also supported by animal model data with accelerated aSyn pathology in the setting of AB amyloidosis (Bassil et al. 2020). Moreover, in vitro cell models demonstrate aSyn ability to cross-seed tau fibril formation (Lee et al. 2004; Guo et al. 2013) and human autopsy data in the Contursi kindred of A53T *SNCA* familial PD have additional substantial tau pathology (Duda et al. 2002b), further highlighting potential synergistic interactions of mixed ADNC/aSyn pathology in the human brain. Thus, it is important to account for ADNC co-pathology when disentangling the biological differences in PDD vs. DLB, which appear to overall represent more of a clinicopathological spectrum than a biological dichotomy (Ballard et al. 2006; Irwin et al. 2017) since, despite these important group-level differences in pathological findings between PDD and DLB syndromes, neuropathological findings alone in an individual patient are insufficient to accurately differentiate these syndromes with high diagnostic accuracy (Irwin et al. 2017; Dickson et al. 2009).

As described further below, higher ADNC pathology postmortem is associated with a shorter MDI in PDD (Irwin et al. 2012, 2013; Sabbagh et al. 2009; Ballard et al. 2006), closer to the 1 year-rule, which currently distinguishes DLB from PDD. Thus, in our comparative autopsy/biomarker studies, we use a 2 × 2 experimental design to both compare and contrast clinical (PD/PDD vs. DLB) and clinically-combined pathologic groupings (i.e. “LBD + AD”= PD/PPD and DLB patients with medium/high-level ADNC vs. “LBD-AB”= PD/PDD and DLB patients with no/low level ADNC), where we find overall stronger associations with biological groups (LBD + AD vs. LBD-AD) than clinical phenotype (PD/PDD vs DLB) alone (Irwin et al. ,^2012^, ^2017^, ^2018^; Coughlin et al.^2019^, 2020b, c; Howard et al. 2021; Shellikeri et al. 2022; Cousins et al. 2023; Cohen et al. 2025; Arezoumandan et al. 2024; Walker et al. 2022; Spotorno et al. 2020). This underscores the strong clinical influence of ADNC on clinical features of LBD, and supports a spectrum of aSyn disorders rather than distinct biological entities defined by the PD/PDD vs. DLB dichotomy. Beyond these autopsy data, there are additional clinical features of shared prodromal states and genetic associations between PD/PDD and DLB that also support unifying these syndromes as a biological construct and spectrum of aSyn disorders.

A major recent advance for clinical criteria for DLB (McKeith et al. 2020; Donaghy et al. 2023) and PD (Berg et al. 2015; Heinzel et al. 2019) is the recognition of early or prodromal symptoms present in functionally-unimpaired individuals, representing the earliest manifestations of aSyn disease. Early diagnosis is critical to improve supports for PD and DLB, as well as to test and implement disease modifying therapies needed to treat the biological cause of these disorders (Postuma 2022). Rapid eye-movement sleep behavior disorder (REM SBD; i.e. sleep disorder with lack of atonia during REM sleep) is an important prodromal state that can precede either PD or DLB syndrome, at times by more than a decade, and correspond to underlying aSyn pathology (including multiple system atrophy, MSA which has a predominance of glial aSyn pathology (Wenning et al. 2022) in most patients (Boeve et al. 1998, 2001; Schenck et al. 1996; Tan et al. 1996; Postuma et al. 2006), even those that do not have clinically-manifest LBD at the time of death (Uchiyama et al. 1995; Maya et al. 2024; Boeve et al. 2007). A large multicenter study estimates roughly a ~ 6–7% annual risk of developing either PD or DLB in sleep polysomnogram-confirmed REM SBD patients (~ 31.3% phenoconverted at 5 years and 62.2% at 20 years) (Postuma et al. 2019). There are few clinical predictors of progression to DLB vs. PD other than subtle abnormalities on cognitive testing in these patients (Joza et al. 2023, 2024). Interestingly, plasma biomarkers of ADNC, particularly ptau_181_, appear to have a more robust prediction for DLB phenoconversion compared to motor PD onset in REM SBD patients (Delva et al. 2025), aligning with postmortem data above on the comparatively high proportion of ADNC co-pathology in most DLB patients.

The pathological substrate for REM SBD is not entirely clear but likely involves cholinergic pontine nuclei and noradrenergic areas of the locus coeruleus (Boeve 2013; Dugger et al. 2012). In the rare autopsy data from isolated idiopathic REM SBD patients mentioned above (Maya et al. 2024; Uchiyama et al. 1995; Boeve et al. 2007), aSyn pathology was found within a relatively restricted distribution largely to pontine nuclei and other brainstem and limbic regions without neocortical involvement and limited co-pathology compared to those REM SBD patients who eventually developed PDD or DLB, potentially consistent with a caudo-rostral spread of aSyn. Since REM SBD can also occur after the onset of PD or DLB, the timing of this and other LBD prodromal features, including anosmia, autonomic dysfunction, mood disorders (including a delirium-onset and psychiatric presentations) could inform pathways of aSyn pathology to compliment the limitations of cross-sectional autopsy-data alone (Fereshtehnejad et al. 2019). Indeed, postmortem examination of the peripheral nervous system in LBD finds variable peripheral aSyn pathology, which is higher for LBD with low-levels of ADNC co-pathology compared to those with high levels of mixed pathology, but interestingly not clearly found in isolation to central nervous system aSyn (Beach et al. 2010). Examination of the autonomic system in living PD patients with or without prodromal REM SBD using multimodal imaging of the cardiac/enteric autonomic system and the dopaminergic system to model aSyn propagation in vivo suggest two main patterns of aSyn spread within the central and peripheral nervous system (Horsager et al. 2020a) which partially overlap with other proposed staging systems (Mastenbroek et al. 2024). These include a top-down unilateral “brain-first” variant with early involvement of the dopaminergic system corresponding to early olfactory and striatonigral involvement compared to an ascending more symmetric “body-first” variant, with early peripheral autonomic involvement prior to the onset of dopaminergic dysfunction, largely corresponding to the caudo-rostral spread of aSyn originally proposed by Braak and colleagues (Horsager and Borghammer 2024 ; Borghammer et al. 2021; Hoager et al. rs^2020^). The DLB phenotype is enriched in body-first subtype including early REM SBD (Borghammer et al. 2021, 2024) suggesting caudo-rostral spread of aSyn could contribute to the DLB syndrome, however, this does not resolve the subset of DLB cases (and clinical AD) with aSyn pathology restricted to amygdala/limbic regions without peripheral involvement (Toledo et al. 2016; Attems et al. 2021; Beach et al. 2009 ), which would correspond more closely to the “brain-first” pathway. Moreover, the brain-first pathway suggests early dopaminergic involvement, but this is also variable in DLB patients with amygdala-predominant aSyn (Toledo et al. 2016). Thus, these models of spread, while informative, likely do not capture the full heterogeneity of aSyn spread within the neuroaxis and do not fully define PDD vs. DLB as a dichotomy. Indeed, further heterogeneity in aSyn topographic distribution and progression is likely within both syndromes, including variable involvement of sympathetic and parasympathetic pathways recently described within the body-first paradigm (Andersen et al. 2025). These intriguing models of aSyn propagation likely highlight the need to discover additional biological factors that influence clinical heterogeneity of LBD beyond these syndromes alone.

Genetic insights also give important biological information for disease pathogenesis in LBDs. First, causal pathogenic variants, including *SNCA* point mutations and gene duplications/triplications are associated with both PD/PDD or DLB syndromes, at times within the same family (Zarranz et al. 2004; Singleton et al. 2003; Chartier-Harlin et al. 2004; Ikeuchi et al. 2008; Polymeropoulos et al. 1997). This shared biologic underpinning in familial forms of LBD may also inform the biological links between PD/PDD and DLB in sporadic disease. Indeed, observations of Parkinsonism along with post-mortem findings of aSyn pathology in adult onset Gaucher’s disease patients led to discovery of *glucocerebrosidase-A (GBA)* pathogenic variants as a risk factor for both PD and DLB (Tayebi et al. 2003; Lwin et al. 2004; Sidransky et al. 2009; Sidransky and Lopez 2012). *GBA* variants are more commonly associated with clinical DLB than PD, with an earlier age at onset, (Sidransky and Lopez 2012), relatively pure neocortical distribution of aSyn (i.e. low/no ADNC co-pathology) postmortem, and more rapid decline (Clark et al.^2009^; Mata et al. 2016; Tsuang et al. 2012). Thus, *GBA*, in addition to ADNC co-pathology, is another important biological contributor to the heterogeneity of LBD. Indeed, clinical trials in *GBA* + PD are targeting this mechanism of lysosomal dysfunction (e.g. NCT06193421). Other lysosomal genes have been linked to aSyn including *PGRN (*Reho et al. 2022) *and GALC (*Hatton et al. 2022), suggesting lysosomal dysfunction may be a shared mechanism in LBD, particularly with pure aSyn. Apolipoprotein (*APOE*) e4 allele, a common risk factor for AD, has been independently linked for risk of aSyn pathology in addition to ADNC co-pathology (Tsuang et al. 2013; Wider et al. 2012; Kaivola et al. 2022). Finally, genome-wide association studies in DLB report novel risk variants, but also overall largely find shared risk between both PD and AD (Kaivola et al. 2022; Chia et al. 2021; Guerreiro et al. 2018) further supporting a spectrum of disease.

In the absence of an in vivo marker to quantify the topology of aSyn pathology in the neuroaxis of living patients, end-stage autopsy data does not preclude the possibility of anatomic subtypes of progression that could define the PDD vs. DLB dichotomy; however, based on above there are likely biological subtypes within and between these syndromes. Another caveat to postmortem work is methodological differences (Beach et al. 2008 ) between studies and the partial discrepancies between the presence of aSyn pathology and neuron loss in some brain regions (Surmeier et al. 2017), necessitating future autopsy work to include digital pathology for more quantitative metrics of multiple aspects of neurodegeneration, including neuron loss (Gawor et al. 2024) and neuroinflammation (Otero-Jimenez et al. 2025; Bachstetter et al. 2015), to help model and contrast the pathologic topology of PD/PDD and DLB. These limitations notwithstanding, there are heterogenous patterns of prodromal features and postmortem aSyn within the neuroaxis in LBD that do not clearly define a dichotomy between PD/PDD vs. DLB, and instead support a spectrum of heterogenous biological subtypes, including genetic risk, across a continuum of LBD and with LBD and AD.

### Clinical influence of ADNC co-pathology and boundaries between LBDs and AD

The presence of ADNC co-pathology has a strong clinical influence on the clinical features of PD/PDD and DLB, both in terms of overall prognosis as well as in specific cognitive and motor features (Coughlin et al. 2020a). Clinically-relevant ADNC pathology (i.e. medium-high level ANDC) is common in LBD (~ 50% overall) and enriched in DLB (~ 70%) compared to PDD (~ 35%) at autopsy (Irwin et al. 2012, 2017; Tropea et al. 2023). PDD patients with higher levels of ADNC tend to have greater overall severity and distribution of cortical aSyn corresponding to a more rapid clinical decline and shorter MDI, often approaching the one-year rule boundary between PDD and DLB (Irwin et al. 2012, 2017; Ballard et al. 2006; Tropea et al. 2023; Halliday et al. 2011). Indeed, overall ADNC co-pathology has an independent effect from age on a shorter time interval to dementia and death across PDD and DLB, and tau NFT pathology specifically is a strong correlate for a poor prognosis (Irwin et al. 2012, 2017). Moreover, the regional distribution and severity of NFTs in LBD influence specific features of cognitive impairment. While core cognitive domains of attention, executive and visuospatial domains characterize PDD (Emre et al. 2007) and DLB (McKeith et al. 2017) (Table 1), ADNC co-pathology (Peavy et al. 2016; Kraybill et al. 2005; Ryman et al. 2021) and particularly the distribution and severity of NFTs in the temporal neocortex and hippocampus correlate with increased difficulty in non-core domains including language impairments and episodic memory in PDD and DLB (Coughlin et al. , 2019). Moreover, motor symptoms of greater postural-instability relative to rest-tremor predominance is a common feature of DLB (Litvan et al. 1998) and poor prognostic sign in PD (Jankovic et al. 1990; Alves et al. 2006) which has also been linked to ADNC co-pathology in PD and DLB (Walker et al. 2022; Kang et al. 2013; Irwin et al. 2020). Finally, increasing ADNC co-pathology is associated with lower frequency and/or severity of core DLB clinical symptoms (Merdes et al. 2003; Kraybill et al. 2005; Ferman et al. 2020). Prospective studies of living patients with in vivo biomarkers of ADNC (Barba et al. 2024; Siderowf et al. 2010; Irwin et al. 2020; Lemstra et al. 2017; Kantarci et al. 2017; Gomperts et al. 2016; Diaz-Galvan et al. 2024), including recent applications of the A/T/N biological framework for AD (Jack et al. 2016) to classify PD (Cousins et al. 2024) and DLB (Ferreira et al. 2020; Jain et al. 2024; van de Beek et al. 2022) patients based on the presence/absence of biomarker evidence of amyloidosis (“A”) and tau NFT pathology (“T”), have found clinical correlations that largely overlap with postmortem observations above, emphasizing the importance of biological characterization of LBDs for clinical research. Indeed, in our previous direct comparison of neuropsychological test performance between biological groups of LBD (Howard et al. 2021), we found worse performance on a language measure of temporal-lobe mediated confrontation naming that we previously found associated to temporal NFT pathology postmortem in LBD (Coughlin et al.^2019^), in LBD + AD compared to LBD-AD, despite similar group-level results between overall PDD and DLB syndrome groups, demonstrating the potential advantage of biological characterization of combined PDD and DLB cohorts for clinical studies over the classic dichotomization of PDD vs. DLB alone.

It is important to note that up to 50% of patients with clinical AD and primary ADNC pathology (Hamilton 2000) (including familial forms *PSN1/2* (Lippa et al. 1998; Leverenz et al. 2006), *APP* (Lippa et al. 1998), Down’s syndrome (Lippa et al. 1999) may have additional aSyn pathology without clinically manifest LBD, representing an additional clinicopathologic spectrum of LBD with AD, further supporting synergy between mixed pathologies. Indeed, postmortem studies of AD patients with additional aSyn pathology find male sex, earlier age at onset, neuropsychiatric and motor Parkinsonism features that do not meet full criteria for DLB are more common compared to AD patients with ADNC without aSyn pathology (Nelson et al. 2010; Chung et al. 2015; Savica et al. 2019). Interestingly, autopsy-confirmed clinical AD with aSyn co-pathology is more likely to have an amygdala/limbic-predominant distribution with relative sparing of the striatonigral system and peripheral nervous system involvement (Toledo et al. 2016; Beach et al. 2010; Mastenbroek et al. 2024). The clinical relevance of amygdala-predominant aSyn is not entirely clear. This relative isolated distribution of aSyn is most commonly associated with clinical AD and advanced stages of ADNC (Uchikado et al. 2006), where is has been linked to antemortem depressive symptoms (Lopez et al. 2006) and cognitive decline in one recent large retrospective study (Almeida et al. 2025). Interestingly, amygdala-predominant aSyn is also found less commonly in a subset of both patients with clinical DLB (Attems et al. 2021) and asymptomatic patients with incidental LBD (Beach et al. 2009). Moreover, aSyn in the amygdala is not uncommon in other neurodegenerative disorders, such as tauopathies (Popescu et al. 2004). Indeed, the amygdala is a region relatively prone to multiple age-related pathologies (Nelson et al. 2018) where aSyn pathology can co-localize with AB plaques, NFTs and TDP-43 inclusions (Nelson et al. 2018; Uchikado et al. 2006; Sorrentino et al. 2019), and correlates with hippocampal neuron loss (Gawor et al. 2024). Finally, aSyn pathology within the amygdala of AD patients with amygdala-predominant pattern is largely in the form of LBs with less neuritic and astrocytic aSyn pathology compared to LBD patients with more widespread aSyn, suggesting strain-like characteristics and other biological factors may contribute to the spread of pathology beyond the amygdala in LBD (Sorrentino et al. 2019). These data highlight the potential synergy of mixed pathology across the clinical spectrum of LBD and AD.

In regards to tau pathology in clinical AD with mixed aSyn pathology, there may be a partially distinct pattern of NFTs compared to patients with clinical LBD (Walker et al. 2015), further highlighting heterogeneity in the topographic patterns of aSyn and ADNC in the neuroaxis beyond clinical PD/PDD vs. DLB. It is tempting to hypothesize that the relative timing of mixed pathologies can influence the clinical expression of aSyn from postmortem data. In a comparative study of patients with comorbid medium-high level ADNC/neocortical aSyn pathology, we found those patients who presented with clinical LBD (PDD or DLB) had a slightly lower burden of amyloid and much lower burden for markers of tau NFT maturity, despite equivalent levels of cortical aSyn, to those who presented with clinical AD, suggesting that earlier or more advanced NFT pathology can influence the clinical expression of mixed pathologies across AD/LBD to a more AD-like phenotype (Arezoumandan et al. 2024). Future work with in vivo biomarkers of AB, tau and aSyn with autopsy confirmation will help resolve the temporal patterns of pathology and resultant phenotype to determine biologically-relevant subgroups of LBD and AD. Indeed, emerging work using in vivo detection of aSyn pathology with SAA from CSF in clinical AD and aging cohorts find associations with greater cognitive decline (Tosun et al. 2024; Quadalti et al. 2023; Palmqvist et al. 2023; Jonaitis et al. 2025), core clinical features of DLB (Quadalti et al. 2023) and AD biomarker positivity (Tosun et al. 2024; Palmqvist et al. 2023; Jonaitis et al. 2025), further suggesting a spectrum of LBD and AD with mixed pathology.

## Emerging biological classification Schemes - Gaps and future directions

Two independent groups recently formulated biologic classification frameworks (i.e. SynNeurGe (Höglinger et al. 2024) and the Neuronal α-Synuclein Disease-Integrated Staging System; NSD-ISS (Simuni et al. 2024a) for LBD based on the presence of genetic and/or biological evidence of aSyn, along with evidence of neurodegeneration and clinical impairment. Comparison of these biological frameworks are summarized in detail elsewhere (Höglinger et al. 2024; Goldman et al. 2025; Höglinger and Lang^2024^), as well as the discussions raised regarding the applicability to all forms of LBD (Simuni et al. 2024c; Lang et al. 2024; Cardoso et al. 2024; Arnaldi et al. 2024; Boeve et al. 2024; Carli et al. 2024; Escamilla-Sevilla et al. 2024; Goldman et al. 2024; Ketchum and Chin 2024; Reis et al. 2024). Of note, these include difficulties in finding a universally applicable measure of neurodegeneration across LBD, considering the findings of relative sparing of the striatonigral system in a subset of DLB. Moreover, these frameworks do not include stratification for ADNC pathology, while recent AA criteria for AD propose a biological definition of AD based on the presence of biological markers of amyloid and two-tiered system of tau pathology, while also incorporating biological measures of aSyn and other age-related pathologies (Jack et al. 2024). Finally, SynNeurGe (Höglinger et al. 2024) also accounts for genetic subtypes of PD which do not have postmortem aSYN, including *LRRK2* variants which are variably associated with aSyn pathology postmortem and ~ 65% of living patients are positive for CSF aSyn SAA (Siderowf et al. 2023). Interestingly, recent work finds SAA positivity from anterior cingulate gyrus in a subset of *LRRK2* autopsy-confirmed cases without aSyn pathology (Garrido et al. 2023). Moreover, *LRRK2* patients who lack overt aSyn pathology with traditional histopathologic methods show oligomeric early forms of aSyn pathology postmortem using a proximity ligation assay (Sekiya et al. 2025). Thus, further study of current and future in vivo assay specificity and sensitivity to all forms of aSyn pathology may enhance biological classification and definitions of LBD.

The shift of biological diagnoses from classic clinicopathological diagnoses formed from retrospective autopsy work is a major advance but not without complexity, challenges and potential opportunities. Indeed, the definition of disease as biological evidence of aSyn and/or ADNC also raises important ethical concerns for use in asymptomatic populations due to the lack of absolute prediction of phenoconversion to a neurodegenerative disease during life in patients who have biological evidence of amyloid, tau and/or aSyn (Parkkinen et al. 2005; Dubois et al. 2024). In reference to the potential of ADNC/aSyn positive testing in an asymptomatic patient to be classified as pathological, perhaps an inference can be made from “super-ager” cohorts whom have high-level cognitive performance in their *≥* 9th decade of life with minimum age-related pathology at autopsy (Weintraub et al. 2025), suggesting these pathologies are not an inevitable result of aging and instead represent a pathologic process, even if not clinically manifest at the time of death. Future work on predictive value of synuclein markers in asymptomatic individuals as well as bioethical research on appropriate disclosure will address these issues. These concerns also raise important context to the use of aSyn markers and AD clinical tests, the former which require additional validation and standardization (Lashuel et al. 2025). Indeed, autopsy validation data is largely limited to CSF SAA, where CSF SAA may have lower sensitivity to detect amygdala/limbic-predominant distributions of aSyn (Arnold et al. 2022) which are more common in DLB or AD with mixed pathology (Toledo et al. 2016; Mastenbroek et al. 2025; Attems et al. 2021). Thus, patients with amygdala-predominant aSyn pathology may be difficult to diagnose with current assays, which currently provide categorical information and are not clearly established as quantitative. Conversely, biofluid biomarkers of ADNC may be influenced by the presence of mixed aSyn pathology (Irwin et al. 2018; Cousins et al. 2022), possibly necessitating disease-specific cut-points in LBD (Weinshel et al. 2022). Therefore, before research frameworks for aSyn can be effectively adapted for clinical use, optimal cut-points, and ideally quantitative metrics of aSyn will be available, that can detect low-levels and anatomically-restricted patterns of aSyn in early prodromal state across LBD and AD.

Despite these potential limitations, efforts to biologically characterize aSyn disorders represent an important step for a precision medicine approach to LBD which can eventually improve clinical trial design and clinical care for patients. Perhaps a nuanced view is that both our classic LBD diagnoses (PD, PDD, DLB) and emerging biologic frameworks play an important role in clinical research in different contexts (Table 3). Importantly, a clinical diagnosis of DLB or PD helps communicate and advocate for appropriate care in these patient population. Moreover, the clinical DLB diagnosis helps differentiate those cognitively impaired patients with a high likelihood of neocortical aSyn pathology from those who do not (i.e. high specificity) (Skogseth et al. 2017; Lopez et al. 1999; Litvan et al. 1998), providing an important high pre-test probability for the use of emerging aSyn biological assays. A critical gap is the lack of an in vivo imaging method to detect the distribution and severity of aSyn pathology, which can help resolve biological differences in the timing and progression of clinical symptoms across the spectrum of PD/PDD, DLB and AD. The dichotomization of PDD vs. DLB within the biological classification of neuronal synucleinopathies facilitates comparative studies needed to discovery of biological and environmental factors that contribute to motor and non-motor neuronal vulnerabilities/protective factors to aSyn. In terms of clinical trial design, symptomatic therapies targeting early motor features in PD or neuropsychiatric/cognitive features of DLB, or later phase III clinical trials focused on clinical outcomes enriched in PD or DLB may benefit from these distinctions as well. Importantly, there is a critical need to develop clinical outcome measures more inclusive of the varied clinical symptoms of all forms of LBD (Rodriguez-Porcel et al. 2022; Toledo et al. 2023; Goldman et al. 2020). Thus, these potential benefits of diagnostic splitting do not negate the importance of increased collaboration across the clinical disciplines of cognitive, movement disorder and autonomic neurology along with psychiatry and sleep medicine to wholistically study the heterogeneous manifestations of aSyn, nor does this limit the importance of increased cross-talk across the fields of AD, PD and DLB clinical and basic research. Ongoing post-mortem validation of aSyn biomarkers is needed across the spectrum of LBD and AD to resolve these issues, and rigorous interrogation of postmortem tissues with digital pathology and molecular techniques in the context of the human brain connectome are needed to discover neuroprotective or vulnerability factors that could further explain phenotypic heterogeneity of aSyn.

**Table 3:** Possible context of use for lumping vs. splitting PDD and DLB clinical diagnoses

“Splitting” PD/PDD vs. DLB into syndromes.	“Lumping” PD/PDD and DLB into a spectrum of aSyn.
Patient care and advocacy, including communication of prognosis, supports and symptomatic therapies for unique needs of DLB or PD patients compared to each other and other age-related neurodegenerative disorders*	Enhance observational studies comparing clinical/biological measures in patients with likely aSyn pathology to other neurodegenerative disorders. This approach can lead to refined clinical classifications of LBD that better reflect biological subgroups of aSyn disorders than PDD/DLB dichotomy alone
Comparative studies of PDD vs. DLB to discover vulnerability/protective factors (e.g. genetic, environmental, patient-specific) for cognition or motor function in aSyn disorders**	Epidemiological studies to detect aSyn disorders and risk factors specific to aSyn pathobiology compared to other diseases
Clinical trials for therapies/interventions with primary clinical endpoints (i.e. Phase III) and/or focused on clinical features enriched in early PD/PDD (e.g. motor) vs. DLB (e.g. cognitive)**	Clinical trials for disease-modifying therapies targeting aSyn (after stratification for ADNC co-pathology), particularly for early phase clinical trials focused on target engagement

* Biological classification of aSyn, once fully validated clinical tests are available, are likely to help facilitate early diagnosis and treatments for both PD/PDD and DLB. DLB clinical syndrome can potentially be helpful to improve pre-test probability of aSyn clinical testing if/when validated for clinical use

**These studies/trials can be enhanced by biological characterization on the presence of aSyn neuropathology, especially as more quantitative measures sensitive to early pathology across all forms of LBD are developed

The biologic frameworks for aSyn and AD are an important first step to comprehensive biologic characterization of patient cohorts, which is critical for advancing clinical trials. Indeed, aSyn-targeted therapeutic trials could potentially include both PDD and DLB patients, particularly after stratifying for the effects of AD co-pathology, *GBA* risk variants, and other important biological factors as these are discovered for biologically-homogenous cohorts. This would be particularly advantageous in early phase trials where target engagement is a main objective and would potentially increase accessibility to trials for PDD patients, particularly those with a short MDI approaching the 1-year rule, who may be potentially excluded from both PD- and DLB-focused trials. Biological stratification also has the potential to improve epidemiologic detection of aSyn disorders, including a high priority to study of the specific effects of sex (Bayram et al. 2025; Nelson et al. 2010) and ethno-racial diversity on the aSyn (Bayram et al 2023; Rizig et al. 2023). The use of a biological framework for aSyn and ADNC has the potential to reduce the ambiguity in the literature on the interpretation of clinical and biological measures reported in non-autopsied LBD cohorts. For example, measures of functional or structural imaging in clinical DLB relate in part to ADNC co-pathology (Spotorno et al. 2020; Nedelska et al. 2015; Graff-Radford et al. 2014; Cohen et al. 2025), as does biological evidence of aSyn on functional imaging in clinical AD (Duong et al. 2025); thus, clinically-defined cohorts of AD or LBD without biological classification do not capture this biologic heterogeneity, and therefore limit interpretation of the data, as these syndromes represent mixed populations of disease.

In conclusion, clinically-impactful therapies in LBD are urgently needed and the opportunity to intervene on pathologic mechanisms as early as possible will require a biologically-based approach. A careful clinical evaluation and our classic clinical phenotypes are important to guide judicious use and interpretation of emerging biological tests for LBD and AD, with the overall goal of early detection and treatment of age-related neurodegenerative pathologies.
